# Gate-tunable charge carrier electrocaloric effect in trilayer graphene

**DOI:** 10.1038/s41598-021-01057-0

**Published:** 2021-11-09

**Authors:** Natalia Cortés, Oscar Negrete, Francisco J. Peña, Patricio Vargas

**Affiliations:** 1 Departamento de Física, Universidad Técnica Federico Santa María, Casilla 110V, Valparaíso, Chile; 2 Centro para el Desarrollo de la Nanociencia y la Nanotecnología, 8320000 Santiago, Chile

**Keywords:** Electronic properties and materials, Quantum physics, Thermodynamics, Electronic properties and devices

## Abstract

The electrocaloric (EC) effect is the change in temperature and entropy of a material driven by the application of an electric field. Our tight-binding calculations linked to Fermi statistics, show that the EC effect can be produced in trilayer graphene (TLG) structures connected to a heat source, triggered by changes in the electronic density of states (DOS) at the Fermi level when external gate fields are applied on the outer graphene layers. We demonstrate that entropy changes are sensitive to the stacking arrangement in TLG systems. The AAA-stacked TLG presents an inverse EC response (cooling) regardless of the temperature value and gate field potential strength, whereas the EC effect in ABC-stacked TLG remains direct (heating) above room temperature. We reveal otherwise the TLG with Bernal-ABA stacking generates both the direct and inverse EC response within the same sample, associated with gate-dependent electronic transitions of thermally excited charge carriers from the valence band to the conduction band in the band structure. The novel *charge carrier electrocaloric effect* we propose in quantum layered systems may bring a wide variety of prototype van der Waals materials that could be used as versatile platforms to controlling the thermal response in nanodevices.

## Introduction

The rapid increase in the need for efficient, environmentally friendly and low-cost materials with the capability of cooling has opened interesting possibilities to explore material properties beyond the traditional vapor-compression method applied in household and industrial refrigeration^1,2^. As a clean and efficient alternative to tuning the systems temperature can be the implementation of caloric effects on solid-state materials, driven by applied pressure, electrical, magnetic and/or mechanical fields, conducting to the barocaloric, electrocaloric (EC), magnetocaloric and elastocaloric effect, respectively^2–4^. Thermal response on solid systems is produced by either of those external field acting on and/or removed from the sample under adiabatic conditions^5^, leading to large temperature changes $\Delta T$ associated with isothermal entropy changes $\Delta S_{T}$, generally near phase transitions^3,6^.

In the last decades, the EC effect—the change in temperature and entropy as an electric field is applied—has been widely studied in three-dimensional dielectric multilayer capacitors (MLC) by considering a different number of layers with diverse thicknesses in the samples, typically of the order of micrometers^7,8^, and finding thickness-dependent thermal responses^8^. In single-crystal MLC, the temperature change reaches $\sim 0.9$ K near ferroelectric phase transitions when moderate electric fields are applied^9^. In high-quality samples of MLC, the temperature change increases up to 5.5 K^6,10^. As films of MLC scales down to hundreds of nanometers, a giant EC response of $\sim 12$ K is manifested close phase transitions^11^. Theory predicts larger cooling power in thin-film MLC of ceramics and polymers by varying the number of layers of the systems^12^. The feasible scalability of MLC systems to reduced dimensionality may allow expanding the EC effect to novel layered structures for electrical refrigeration at the atomistic level^13,14^.

Nanoscale van der Waals (vdW) multilayers and heterostructures comprise a wide variety of quantum solids^15^ that could be used as prototype miniaturized material systems to get caloric effects^16^. A giant EC response of $\sim 23$ K has experimentally been achieved in a ferroelectric heterobilayer because of interface-induced interactions between the constituent materials^17^. Theoretical studies on graphene nanoribbons under an electric and magnetic field show controllable entropy changes due to the applied magnetic field^18^. It has been reported that a mechanical strain can produces entropy changes in a graphene monolayer^19^. In a Bernal bilayer graphene an oscillating EC and magnetocaloric effect is obtained^20^. First-principle and thermodynamic calculations in a two-dimensional (2D) monolayer of MoTe$_2$, demonstrated a temperature change of 10–15 K near the structural phase transition that occurs when the monolayer is subject to electrostatic gating^21^.

We explore here the thermal response of atomically thin vdW nanostructures, focusing on gated trilayer graphene (TLG) systems. Graphene multilayers can experimentally be obtained with different stacking orders depending on the horizontal shift between consecutive graphene planes, leading to distinct TLG topology and thus an unique electronic structure fingerprint for each TLG sample. Consequently, diverse experiments and theory have revealed that the electronic band structure and density of states (DOS) strongly depend on the stacking pattern they possess, showing significant changes around the Fermi energy when electrical potentials are applied on the TLG systems^22–30^. Through the novel EC phenomenon due to charge carriers in vdW systems we propose here, we are joining the physics of quantum layered 2D materials—using the electronic band structure and DOS—with thermodynamics, so that the electronic entropy plays a fundamental role, which we demonstrate its fully dependent on the stacking arrangement of each TLG because of its distinctive topology. We are interested in the TLG DOS in the vicinity of the Fermi level, so that we can expect different entropy responses because of the stacking-dependent electronic structure that possess each TLG. This makes TLG materials suitable to inspect the EC effect as the electronic entropy can be altered with changes of the DOS driven by electrical potentials. Through Bloch electrons in parameterized tight-binding (TB) models linked to Fermi statistics, we show how the thermal response varies within each TLG stacking as a result of the quantum-thermodynamic processes involved at low energies. In particular, for ABA stacked TLG, we unveil the direct connection between the electronic entropy and dual (heating and cooling) EC effect through calculations of gate-dependent thermally excited carriers from the valence band to the conduction band at low and high temperatures. This procedure allows us to predict that for ABA TLG subjected to moderate gate fields, charge suppression (nonlinear excited carriers) can be associated with the heating process of the EC effect, while linear excited carriers contribute to cool down the ABA TLG system.

## Quantum-thermodynamic model

**Figure 1:**
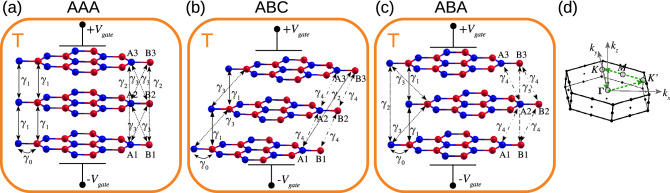
Schematic representation of trilayers graphene with diverse stacking patterns, hexagonal AAA (**a**), rhombohedral ABC (**b**) and Bernal ABA (**c**). Blue (red) sphere show *A* (*B*) carbon sublattice connected through intralayer $\gamma _0$ and interlayer hoppings $\gamma _1$, $\gamma _2$, $\gamma _3$, $\gamma _4$ and $\gamma _5$ as indicated; $\pm V_{gate}$ is the gate potential applied on the outer graphene layers for each system, the orange square enclosing each structure represent the thermal source at temperature *T*. (**d**) Shows the Brillouin zone in momentum space for all trilayers graphene, where green lines highlight the triangular $\varvec{k}$-path where the DOS is calculated with corners located at high symmetry points $\Gamma =(0,0)$, $K=\frac{2\pi }{a}\big (0,\frac{2}{3}\big )$ and $K'=\frac{2\pi }{a}\big (\frac{\sqrt{3}}{3},\frac{1}{3}\big )$, $a=\sqrt{3}a_{\text {C-C}}=2.46$ Å is the 2D graphene lattice constant.

We simulate TLG structures with simple hexagonal AAA, Bernal ABA, and rhombohedral ABC stacking order by using a $\pi$-orbital tight-binding (TB) model that captures the main low-energy symmetry properties for each TLG system^23–25,31–35^. These TB Hamiltonians have also been proved to well support optical conductivity and band structure calculations as compared to experiments^27,36^. To construct the TB Hamiltonians, we consider the unit cells of the TLG with three pairs of carbon atoms {*A*1–*B*1}, {*A*2–*B*2} and {*A*3–*B*3}, which are located respectively in the bottom, central and top graphene layers, as shown in Fig. 1, see “Methods” for the TB model of each TLG. We use different parameterization (hoppings) depending on the stacking type of the TLG, reliably reproducing the first-principle energy dispersion for both cases the unperturbed^37^ (see “Methods”) as well as the perturbed (in the presence of an electric field) system^22,30^.

For each TLG system, we have first taken a minimal set of parameters from fitting photoemission spectroscopy spectra to the TB models^36^, which accurately account for the low-energy spectrum of each stacked TLG. These parameters include a strong intralayer hopping $\gamma _0$ between *Ai* and *Bi* nearest neighbors within each graphene layer ($i=1,2,3$ is the layer index), and one interlayer hopping connecting direct vertical nearest-layer carbon sites with strong coupling $\gamma _1$, see Fig. 1. For ABC and ABA-stacked TLG, this parameterization also incorporates a weaker nearest-layer coupling $\gamma _3$, Fig. 1b,c, respectively. In particular for ABA-TLG, we also have taken a full set of parameters adopting the Slonczewski–Weiss–McClure parameterization^24,38^, which contains one extra nearest-interlayer hopping $\gamma _4$ as well as two next-nearest interlayer hoppings $\gamma _2$ and $\gamma _5$. This latter parameterization has been used in electronic transport experiments, where additional characteristics in the energy spectrum not present in the minimal TB model were identified^39^. The “Supplemental Material” contains the full parameterization for AAA and ABC TLG as shown in Fig. 1a,b, respectively.

An asymmetric onsite-potential energy difference with magnitude $2V_{g}$, induced by a bias voltage between the external graphene layers can act as an external gate potential on each carbon atom of top $+V_{g}$ and bottom $-V_{g}$ graphene layers of the TLG systems, in a similar way to a bilayer graphene^40^ and trilayer structures under an external voltage^41,42^. This asymmetric potential is a crucial quantity to tune the electronic DOS and to produce a caloric response for the TLG systems.

The DOS $D(E,V_g)$ depends of both, the electronic level with energy *E* and the gate potential $V_g$ applied on the outer carbon atoms of each TLG, and allows us to calculate the electronic thermodynamics properties of the TLG systems. As the TLG structures can be considered as quasi 2D systems, we numerically calculate the DOS using a 2D Brillouin zone (BZ) (i.e., $k_z=0$) in reciprocal $\pmb {k}$-space. We use a fine mesh of about ten million of $\pmb {k}$ points in the area enclosed by the green triangle of Fig. 1d, and for every $\pmb {k}$-state we evaluate the energy levels coming from each band of the TLG TB Hamiltonian given in “Methods”. By calculating the DOS, we can obtain the number of electrons *N* in each unit cell of the trilayers

1 $$\begin{aligned} N(V_g,T,\mu )=\int _{E_l}^{E_h}D(E,V_g)n_\text {F}(E,T,\mu )dE, \end{aligned}$$

where $E_{l(h)}$ is the lowest (highest) electronic energy eigenvalue of the considered TLG Hamiltonian, $n{_{\text {F}}}(E,T,\mu )=1/[e^{\beta (E-\mu )}+1]$ is the Fermi-Dirac function distribution with $\beta =1/k_{\text {B}}T$, $k_{\text {B}}$ is the Boltzmann constant, $\mu$ is the chemical potential and *T* the heat-source temperature applied on the TLG systems, schematized with an orange square in Fig. 1. At $T=0$ K, the electronic levels are occupied up to the Fermi energy $E_{\text {F}}$, and $n{_\text {F}}$ it converts in the Heaviside function, so that we obtain $N=\int _{E_l}^{E_{\text {F}}}D(E,V_g)dE=6$ for each TLG unit cell. For finite temperatures $T>0$, we can calculate the chemical potential $\mu (V_g,T)$ by inversion of Eq. (1) and obtain *N*.

The total entropy of the system $S_{\text {total}}=S_{\text {latt}}(T)+S_{e}(V_g,T)$ includes two terms, the entropy of the lattice $S_{\text {latt}}(T)$, giving account of the phonon contribution, in which we assume it is only dependent on temperature and not on $V_{g}$^43^. In our approximation we assume that $S_{\text {latt}}(T)$ vanishes and use $S_{\text {total}} \sim S_{e}(V_g,T)$, where the electronic entropy

2 $$\begin{aligned} S_{e}(V_g,T)=-k_{\text {B}} \int _{E_l}^{E_h}D(E,V_g){\mathscr {F}}(n_{\text {F}})dE, \end{aligned}$$

depends on the gate potential and temperature, and is calculated in the triangular area of the BZ of Fig. 1d, where the number of electrons is fixed to $N=6$. In Eq. (2),

3 $$\begin{aligned} {\mathscr {F}}(n_{\text {F}})=n_{\text {F}}\ln n_{\text {F}}+(1-n_{\text {F}})\ln (1-n_{\text {F}}), \end{aligned}$$

is approximated by a Lorentzian-like function $L(E,T,\mu )=C/[e^{(|E-\mu |/2k_{\text {B}}T)^{3/2}}+1]$. By considering low and high *T* values and $C=1.4$, we obtain excellent agreement between Eq. (3) and $L(E,T,\mu )$, with $-{\mathscr {F}}(n_{\text {F}})\approx L(E,T,\mu )$ (see “Methods” for more details), so that the electronic entropy in Eq. (2) transforms as

4 $$\begin{aligned} S_{e}(V_g,T)\simeq k_{\text {B}} \int _{E_l}^{E_h}D(E,V_g)L(E,T,\mu )dE. \end{aligned}$$

The main contribution of $L(E,T,\mu )$ to the electronic entropy is given by their temperature-dependent width (see Fig. 10 of “Methods”), capturing more available states of the DOS as temperature increases. Within the range of temperature we work, $k_{\text {B}}T \ll \gamma _1$, the chemical potential remains constant at $\mu =0$ eV in order to fulfill $N=6$ for each TLG unit cell, so that we use $\mu =E_{\text {F}}=0$ for all the calculations.

Typically there are two ways to measure the caloric effects in solid materials: (1) through the variation of temperature in an adiabatic thermodynamic path $\Delta T$ (direct measurement), and (2) by means of the entropy change in an isothermal path $\Delta S_{T}$ (indirect measurement), where the subindex *T* indicates constant temperature. It is important to note that is experimentally challenging to obtain $\Delta T$ as compared to $\Delta S_{T}$, as direct measurements generally require precision calorimetry^1^.

The EC effect in our TLG structures is then obtained through the indirect way, by means of electronic entropy difference calculations between the entropy at zero gate field $S_{e}(V_{g}=0,T)$ and a final gate field $S_{e}(V_{g},T)$, and taking both entropies at temperature *T* and $\mu =E_{\text {F}}=0$, this reads

5 $$\begin{aligned} -\Delta S_{e,T}(V_{g},T)= S_{e}(V_{g}=0,T)-S_{e}(V_{g},T). \end{aligned}$$

In case to obtain $-\Delta S_{e,T}>0$, we are in presence of the direct EC effect, that is the TLG system is capable to heat as $V_g$ increases. In the opposite case when $-\Delta S_{e,T}<0$, the system present an inverse EC effect and the sample cools down.

## Results

### AAA-stacked TLG

The AAA-stacked TLG has been recently exfoliated^36^, preserves metallic character in the presence of external electric fields^44^ as in the monolayer graphene case, and possesses mirror reflection symmetry with respect to the central layer. In this structure the {*Ai*, *Bi*} sublattices match to the nearest-neighbor layer carbon sites {$Ai+1$, $Bi+1$} with vertical hopping $\gamma _1$. The whole system effectively can be seen as a direct superposition of three graphene monolayers as shown in Fig. 1a.

**Figure 2:**
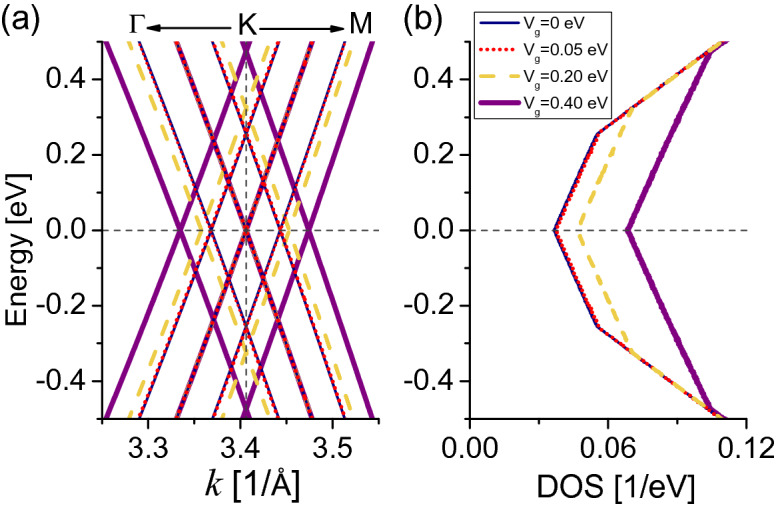
Low-energy electronic spectra for AAA-stacked trilayer graphene. (**a**) Band structure in the vicinity of the *K* point. (**b**) Density of states obtained for the triangular area enclosed by green lines in Fig. 1d. Vertical dashed line in (**a**) highlight the *K* point, horizontal dashed lines in (**a**, **b**) are fixed at $E_{\text {F}}=0$ eV. The hopping parameters are $\gamma _0=3.2$ eV and $\gamma _1=0.18$ eV^36^.

The low-energy electronic structure near the *K* (or $K'$) point considering $\gamma _0$ and $\gamma _1$ couplings and different values of the gate potential $0\le V_g \sim 2\gamma _1$ is presented in Fig. 2a. Due to the structural symmetry, the Dirac points *K*, $K'$ are degenerate, where around each one of them there are three pairs of linear branches creating a diamond structure with Dirac cones throughout the energy-momentum space^44^. At the Fermi level $E_{\text {F}}=0$ and momentum *K*, or at the charge neutrality point (CNP), one of the Dirac cones remains constant regardless of the $V_g$ strength, resembling the monolayer graphene modes.

When $V_g=0$ (thin dark blue solid lines), a pair of Dirac cones shift away from the CNP by $E=\pm \sqrt{2}\gamma _{1}\simeq 0.25$ eV because of interlayer interaction $\gamma _1$. As $V_g$ is turned on, a pair of cones symmetrically disperse to energies $E=\pm \sqrt{V_g{^2}+2\gamma _{1}^{2}}$. The effect of the gate potential therefore can be represented as a renormalization of the interlayer hopping energy^45^, preserving metallic character for the bands as in the unperturbed case (i.e., $V_g=0$). At low-energies, the DOS in Fig. 2b preserves electron-hole symmetry [$D(E,V_g)=D(-E,V_g)$], and shows a global minimum at the CNP, which causes the major contribution to the EC response at low and high temperatures as we describe below. The DOS also shows two symmetric discontinuities at the energies given by the shifted Dirac cones, not providing states to the EC effect as the *L* function in Eq. (4) does not capture those states up to room temperature.

**Figure 3:**
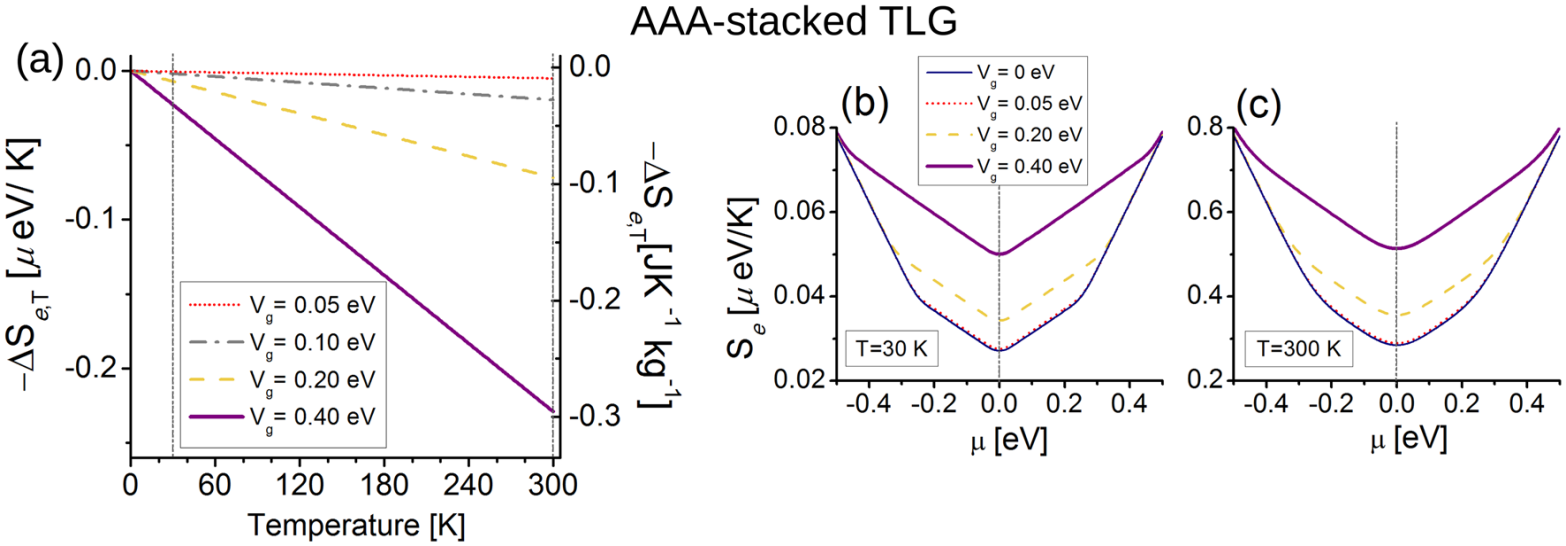
Electrocaloric response for AAA-stacked trilayer graphene. (**a**) Isothermal entropy changes $-\Delta S_{e,T}$ as a function of *T*, the chemical potential is set to $\mu =0$. Dashed vertical lines indicate $T=30$ K and $T=300$ K, where we calculate the electronic entropy in (**b**) and (**c**) respectively. Vertical dashed lines in (**b**) and (**c**) indicate $\mu =0$ eV where we obtain the EC effect in (**a**). Note distinct vertical axes in (**a**) indicating different electrocaloric units.

The EC effect (or $-\Delta S_{e,T}$) for the AAA-stacked TLG is presented in Fig. 3a. The $-\Delta S_{e,T}$ response is linear as a function of *T*, following the linear behavior of the electronic spectra near $E_{\text {F}}$ in Fig. 2. The EC effect is inverse for the entire temperature range from 0 to 300 K, this means that $S_{e}(V_{g}=0,T) < S_{e}(V_g\ne 0,T)$. Hence we always obtain $-\Delta S_{e,T}<0$ from Eq. (5), and the EC effect reaches a value of $\simeq -0.23$ $\upmu$eV K$^{-1}$ ($\simeq -0.3$ J K$^{-1}$ kg$^{-1}$) at room temperature for moderate gate voltage ($V_g=0.4$ eV). These results are comparable to entropy change values of ceramic ferroelectric materials^43,46^. Notice that the inclusion of next-nearest layer (NNL) hoppings does not qualitative change the EC results, see “Supp. Mater.”.

The entropy change results can be explained directly from the plots of the electronic entropy $S_{e}$ (Eq. 4) at $\mu =0$, presented for $T=30$ K in Fig. 3b and $T=300$ K Fig. 3c. $S_{e}$ at $T=30$ K almost preserves the DOS shape of Fig. 2b as the *L* function slowly broadens the DOS at low temperature, while the entropies are largely smoothed at $T=300$ K, with one order of magnitude higher than $T=30$ K. $S_{e}$ at $\mu =0$ and $V_{g}=0$ for both temperatures are the smallest entropies with respect to the other entropies with larger $V_g$, then the AAA-stacked TLG cools down for all $V_g$ we have considered here, as expected from the EC results in Fig. 3a.

### ABC-stacked TLG

Rhombohedral ABC-stacked trilayer graphene is one of the commonly stable crystal obtained in experimental procedures^27,36,47^, it can be considered as a zero-gap semiconductor material in the unperturbed case ($V_g=0$), and as a semiconductor when external electric-field potentials are applied on the sample^22^. The ABC stacking geometry possesses inversion symmetry, but lacks mirror symmetry^27^, as schematized in Fig. 1b.

**Figure 4:**
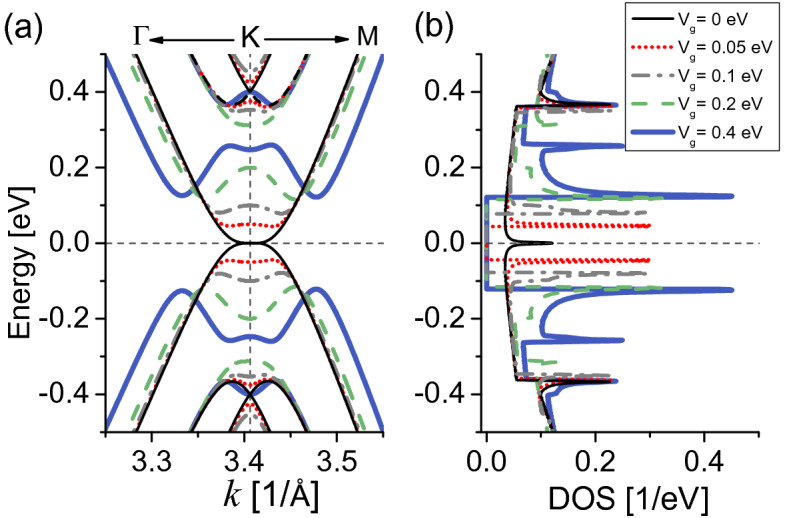
(**a**) Band structure near the *K* point, (**b**) DOS from the green triangle area in Fig. 1d for ABC-stacked trilayer graphene with diverse gate potential $V_g$ as indicated. Horizontal (vertical) dashed lines indicate $E_{\text {F}}=0$ eV (*K* point). The hopping parameters are $\gamma _0=3.10$ eV, $\gamma _1=0.4$ eV and $\gamma _3=0.2$ eV^36^.

The band structure and DOS are plotted in Fig. 4a,b respectively, considering interlayer hoppings $\gamma _1$ and $\gamma _3$, and gate potentials in the range $0\le V_g \le \gamma _1$. The electronic spectra preserve electron-hole symmetry around $E_{\text {F}}=0$ for all $V_g$, notice that this symmetry is broken in the presence of NNL hoppings, see “Supp. Mater.”. In the electronic structure there are valence and conduction bands dispersing according to the hoppings between the different atoms. For $V_g=0$ (thinnest black lines), the lower conduction band and higher valence band touch at $E_{\text {F}}$ near the *K* point, where the DOS shows a local maximum.

Two other pairs of bands shift away from $E_{\text {F}}=0$ and are degenerate, crossing near $E\simeq \pm 0.4$ eV because of the strongly interlayer coupling $\gamma _1$ between the direct bonded {*B*1–*A*2} and {*B*2–*A*3} dimers of the ABC TLG, see Fig. 1b. The DOS increases at these crossing-band energies and presents a pair of local sharp peaks, providing states to the EC effect when the temperature is $T \gg 300$ K. When $V_g$ is turned on, an energy gap between the lower electron (conduction band) and higher hole branch (valence band) opens at the *K* point as well as in their vicinity because of the potential energy difference between the bottom and top graphene layers^22,25,33,37,44,48^. The gaps nonlinearly increase as $V_g$ increases^30,44^, the DOS vanishes for the gap energies as expected, and shows two high symmetric peaks (van Hove singularities) near the valence and conduction band edges, whose states are mainly contributed by the carbon atoms of the top and bottom graphene layers^30^. The bandgap edges for low-gate potential $V_g=0.05$ eV ($V_g=0.1$ eV) provide states to the EC effect for temperatures starting from $\sim 80$ K (140 K), while when moderate gate fields $V_g=0.2, 0.4$ eV are applied, the electronic states start to contribute to the EC response when $T >240$ K.

**Figure 5:**
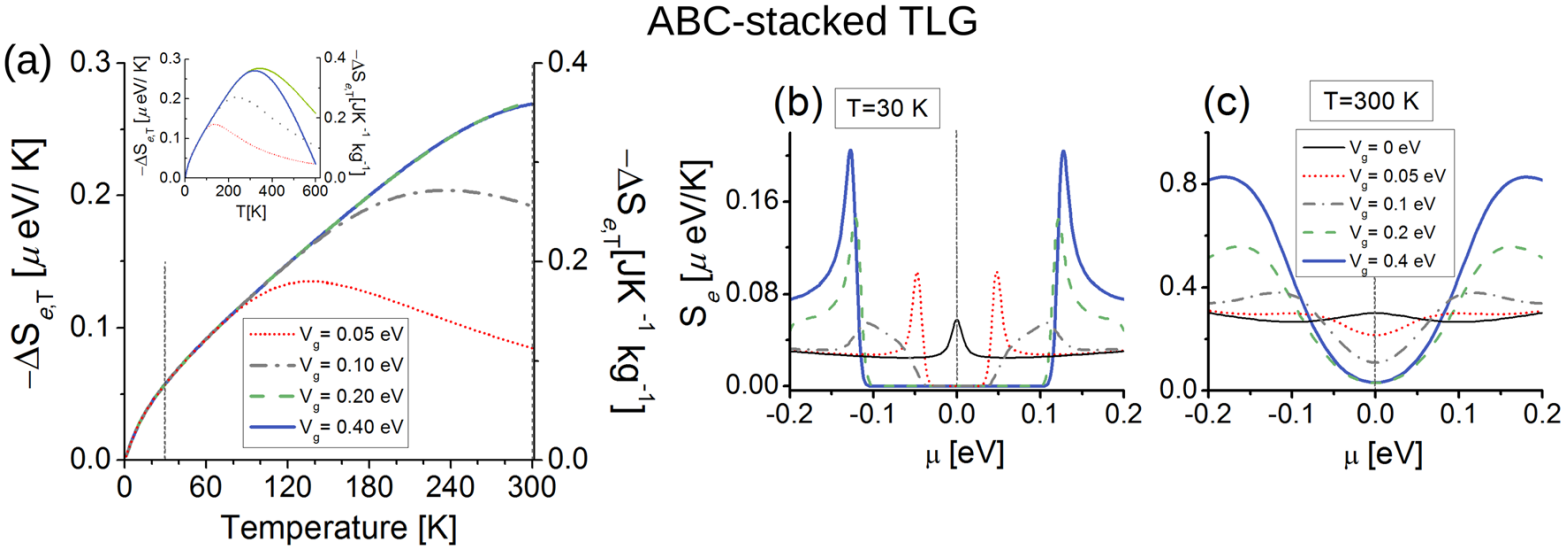
Electrocaloric response for ABC-stacked trilayer graphene. (**a**) Isothermal entropy changes $-\Delta S_{e,T}$ as a function of *T*, the chemical potential is set to $\mu =0$. Dashed vertical lines indicate $T=30$ K and $T=300$ K, where we calculate the electronic entropy in (**b**) and (**c**) respectively. The inset in (**a**) shows $-\Delta S_{e,T}$ for higher temperatures. Note distinct vertical axes in (**a**) indicating different electrocaloric units. In (**b**) and (**c**) vertical dashed lines indicate $\mu =0$ eV where we obtain the EC effect in (**a**).

Figure 5a shows the EC effect for ABC-TLG structure. Here we observe a nonlinear direct response (heating) for temperatures up to 300 K and the gate potentials considered here ($V_g \le 0.4$ eV). This occurs because the DOS evaluated at $D(E_{\text {F}}=0,V_g=0)$ present a maximum [see Fig. 4b], whose states are fully captured by the *L* function at low and high temperatures, while the DOS for $V_{g}\ne 0$ vanishes around $E_{\text {F}}=0$ due to the band gap, hence the *L* function does not capture any state when $V_{g}\ne 0$, and the reference electronic entropy [i.e., $S_e(V_g=0)$] is higher than all the other gate-dependent entropies $S_{e}(V_{g}=0,T) > S_{e}(V_g\ne 0,T)$, see “Supplemental Material” for the ABC EC effect with NNL hoppings. We also have checked that there is not a direct-inverse transition [$-\Delta S_{e,T}=0$] up to $T=600$ K for $V_g \le 0.4$ eV [see inset of Fig. 3b]; however, we found that $-\Delta S_{e,T}=0$ for $V_g>0.4$ eV and $T \gg 300$ K.

In addition, an interesting effect occurs for temperatures below 80 K for all $V_{g}$, in which $-\Delta S_{e,T}$ does not present differences in their caloric responses, we attribute this result to the presence of the band gap in the electronic dispersion. The maxima of $-\Delta S_{e,T}$ for low gate fields as temperature increases correspond to $-\Delta S_{e,T}(V_{g}=0.05\ {\text {eV}}, T=137\ {\text {K}})\simeq 0.14$ $\upmu$eV K$^{-1}$ $\simeq 0.19$ J K$^{-1}$ kg$^{-1}$, and $-\Delta S_{e,T}(V_{g}=0.1\ {\text {eV}}, T=236\ {\text {K}})\simeq 0.20$ $\upmu$eV K$^{-1}$ $\simeq 0.27$ J K$^{-1}$ kg$^{-1}$, these values are comparable to the magnetocaloric response of graphene-based superlattices^49^. The maxima for moderate $V_g$ reach $-\Delta S_{e,T}(V_{g}=0.2\ {\text {eV}}, T=343\ {\text {K}})\simeq 0.37$ $\upmu$eV K$^{-1}$ $\simeq 0.5$ J K$^{-1}$ kg$^{-1}$, and $-\Delta S_{e,T}(V_{g}=0.4\ {\text {eV}}, T=321\ {\text {K}})\simeq 0.36$ $\upmu$eV K$^{-1}$ $\simeq 0.49$ J K$^{-1}$ kg$^{-1}$, see inset of Fig. 5a.

These behaviors are consistent with $S_{e}$ at $\mu =0$ for $T=30$ K in Fig. 5b and $T=300$ K Fig. 5c. For both temperature values, $S_{e}$ fulfills the condition $S_{e}(V_{g}=0,T) > S_{e}(V_{g}\ne 0,T)$ at $\mu =0$, giving the direct EC response, and the ABC-TLG sample heats as expected from $-\Delta S_{e,T}$ results, opposite to the AAA-TLG case. $S_{e}$ at $T=30$ K in Fig. 5b, shows the only entropy contributing to the EC effect in the vicinity of $\mu =0$ is that for $V_{g}=0$, while the other entropies when $V_{g} \ne 0$ vanish and do not provide states to the EC effect. For temperatures lower than 80 K, we have verified that $S_{e}(V_{g}=0,T)\simeq -\Delta S_{e,T}(V_{g}\ne 0,T)$ because of the band gap. At room temperature in Fig. 5c, the electronic entropies are nearly five times larger than the entropies at $T=30$ K, showing non null values for all $V_g$ in the gap region due to larger temperature values. $S_{e}$ is almost flat for $V_g=0$, while the other entropies have a parabolic shape as $V_g$ increases, with almost the same values for moderate gate-potential fields $V_g=0.2,0.4$ eV near $\mu =0$, as expected from the EC response in Fig. 3b at $T=300$ K.

### ABA-stacked TLG

Trilayer graphene with Bernal-ABA stacking is the most stable geometry as resembles a graphitelike atomic structure^22^. The ABA TLG possesses mirror symmetry with respect to the middle graphene layer. This structural symmetry is broken in the presence of electrostatic potentials^24,48^, driving to semimetallic character with tunable overlap of the electronic bands^22,26,30^, as well as controllable gaps at the *K* point and near it^23,30,44^. These band gaps are of fundamental importance to describe the dual cooling and heating process of the EC effect as we will see later. We analyze first a simple case for ABA TLG, labeled nearest-layer (NL) TB model, including hopping parameters $\gamma _0$, $\gamma _1$ and $\gamma _3$, with $\gamma _2=\gamma _4=\gamma _5=0$. We take different values of the external gate potential for the electronic spectra and EC calculations, in the limit $0\le V_g\gtrsim \gamma _1$.

**Figure 6:**
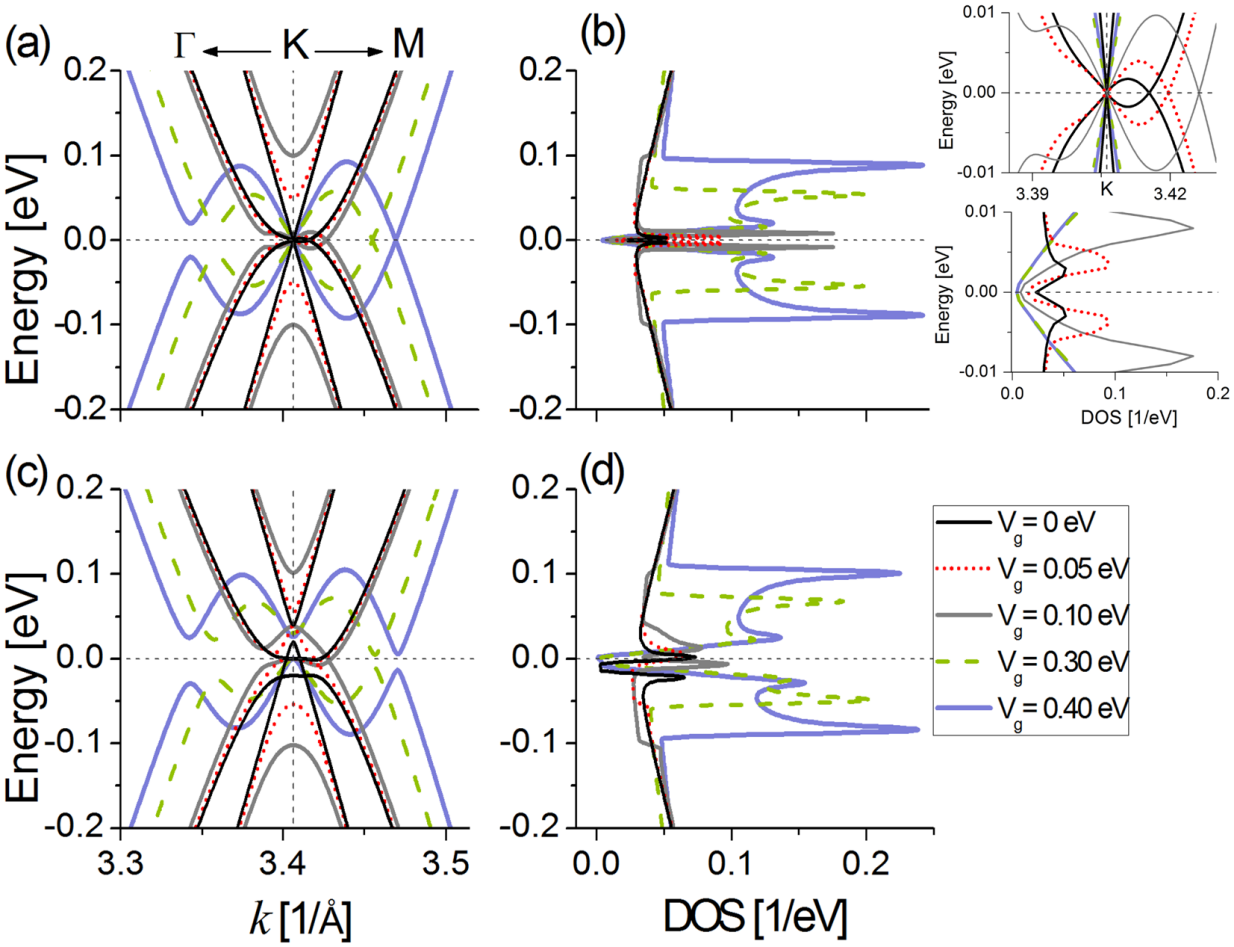
Electronic spectra for ABA-stacked trilayer graphene near the Fermi level using the nearest layer NL (**a**, **b**) and next-nearest layer NNL (**c**, **d**) TB models. Left panels show the electronic band structure about the *K* point in the Brilloin zone. Right panels illustrate the density of states for the triangular area enclosed by green lines in Fig. 1d. (**a**, **b**) $\gamma _0=3.15$ eV, $\gamma _1=0.39$ eV and $\gamma _3=0.25$ eV^36^. (**c**, **d**) $\gamma _0=3.16$ eV, $\gamma _1=0.39$ eV, $\gamma _2=-0.020$ eV, $\gamma _3=0.315$ eV, $\gamma _4=0.044$ eV and $\gamma _5=-0.04$ eV^33^. Horizontal (vertical) dashed line indicates $E_{\text {F}}=0$ (*K* point). Inset in (**b**) shows a zoomed area for the spectra of the NL-TB model (**a**, **b**) around $E_{\text {F}}=0$.

Figure 6a shows the electronic band structure around the *K* point, Fig. 6b corresponds to the DOS. Both quantities are shown in the vicinity of $E_{\text {F}}=0$. Whitin the NL-TB model, the DOS is symmetric about zero energy with and without the presence of the gate field, preserving electron-hole symmetry^24^. For $V_g=0$ (solid black lines), two bands mimic the *k*-linear electronic dispersion of monolayer graphene at the *K* point, and one pair of parabolic bands overlap along the *KM* *k*-axis near $E_{\text {F}}$, see inset of Fig. 6b. The whole unperturbed bands (i.e., when $V_g=0$) give a superposition of states of monolayerlike and Bernal-bilayerlike graphene states^31,32,48^. The DOS shows a sharp minimum at $E_{\text {F}}$ with two symmetric peaks surrounded it [see inset in Fig. 6b]. The major contribution of the edge peaks its from the AB-bilayerlike electron and hole band edges, whose states are fully captured by the *L* function for $T>10$ K.

The gate field breaks mirror reflection symmetry with respect to the central layer and mixes the linear and parabolic bands in the region near $E_{\text {F}}$^24,48^, so that when $V_g=0.05$ eV (red dotted lines) and $V_g=0.1$ eV (gray solid lines), the monolayerlike bands shift from $E_{\text {F}}=0$ and it convert into parabolic bands, whereas the bilayerlike bands remain almost unperturbed. The DOS for these cases presents a minimum at $E_{\text {F}}$, and shows large electron and hole edge peaks as more states are overlapped near $E_{\text {F}}$, contributing to the EC effect for temperatures $T>30$ K. When the gate potential increases comparable to $\gamma _3 \sim V_g =0.3$ eV (dashed green lines) and $\gamma _1 \simeq V_g =0.4$ eV (solid purple lines), linear dispersion is seen at the *K* point as well as along the *KM* direction near $E_{\text {F}}$, while a gap opens along $K\Gamma$ as $\gamma _3$ is non zero^23,36^. For these moderate gate field potentials, the DOS almost vanishes at $E_{\text {F}}$ and shows one pair of symmetric peaks centered near the gap edges, contributing to the EC response when $T \ge 70$ K. Other pair of peaks are centered in the vicinity of the minima of the highest valence band and maxima of the lowest conduction band, not providing states to the EC effect for $T < 170$ K.

When all the interactions between the atoms are taken into account in the ABA TLG system, labeled NNL-TB model, the energy spectra are no longer electron-hole symmetric about $E_{\text {F}}$ because of the additional electron hoppings. However, the full band structure for $V_g=0$ can still be considered as a combination of the band diagram of a single graphene layer and AB-bilayer graphene as shown in Fig. 6c. Furthermore, the valence and conduction parabolic bands open a gap near $E_{\text {F}}$ at the *K* point, where the DOS shows a flat minimum in the hole zone ($E_{\text {F}} \lesssim 0$), Fig. 6d. The electron and hole peak edges from the AB-bilayerlike bands in this case provide states to the EC effect at higher temperatures than in the NL case, $T>60$ K.

In the NNL-TB model, a gate potential of $V_g=0.05$ eV causes anticrossing of the parabolic bands, similar as seen when negative and positive low-charged gates act on a suspended ABA TLG^23^. At $E_{\text {F}}=0$ the DOS increases, showing nearly a maximum instead of a minimum as in the NL case. The electronic states belonging to this maximum are fully captured by the *L* function for $T>30$ K. For $V_g=0.1$ eV, the bands are similar to the NL-TB model, but opening a gap at the *K* point, and showing a highly antisymmetric DOS, whose states near $E_{\text {F}}$ contribute to the EC response for $T>60$ K. Moderate gate potentials $V_g=0.3$ eV and $V_g=0.4$ eV open gaps at the *K* point as well as along both $K\Gamma$ and *KM* *k*-axis, similar to the effect that produces a gate-induced high charge density on ABA TLG^23^. The DOS for these moderate $V_g$ values show minima at zero energy as in the band structure there are allowed states only at the *K* point, contributing to the EC response for $T \ge 30$ K. As we will see below, these band gaps are responsible for the charge carrier EC effect.

**Figure 7:**
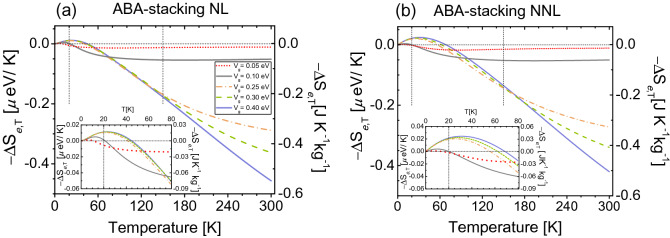
The isothermal entropy changes $-\Delta S_{e,T}$ as a function of temperature *T* for different gate potentials as indicated. (**a**) ABA-stacked trilayer graphene considering the NL-TB model, (**b**) ABA-stacked trilayer graphene with NNL-TB model. The chemical potential is set to $\mu =0$ eV. Horizontal dashed line indicates $-\Delta S_{e,T}=0$, while vertical dashed lines stand for $T=20$ K and $T=150$ K, where we calculate the entropies as a function of $\mu$ in Fig. 8. Insets show a zoom for each ABA-EC response at relatively low temperatures. Note distinct vertical axes indicating different EC units.

In Fig. 7a,b, we respectively present the EC response for both the NL-ABA and NNL-ABA TB model. Both cases are qualitatively similar as they show a dual behavior seen as a combination of the direct and inverse EC effect, whose gate-dependent inversion $-\Delta S_{e,T}(V_{g},T)=0$ occurs at relatively low temperatures. This dual process is not present in other graphene-based systems^49^, with perhaps more practical alternatives in the current system. In both ABA-TB cases we analyze here, we observe a small direct response zone (heating) for low gate-fields $V_{g}=0.05$ eV (dotted red lines) and $V_{g}=0.1$ eV (solid gray lines), with maxima reaching $-\Delta S_{e,T} \le 0.005$ $\upmu$eV K$^{-1}$ $\simeq 0.0067$ J K$^{-1}$ kg$^{-1}$ at $T\le 12$ K—see insets of Fig. 7. As temperature increases for these low-gate field values, $-\Delta S_{e,T}$ become inverse for $T \le 21$ K, then the system constantly cools down up to $T=300$ K.

The EC effect for moderate fields $V_{g}=0.25,0.3,0.4$ eV is very different as compared to low-gate field values $V_{g}=0.05,0.1$ eV as seen in both ABA-TB systems. For the ABA-NL case in Fig. 7a, the maxima of $-\Delta S_{e,T}$ are almost constant, reaching $-\Delta S_{e,T} \simeq 0.011$ $\upmu$eV K$^{-1}$ $\simeq 0.015$ J K$^{-1}$ kg$^{-1}$ at $T \simeq 23$ K. The temperature of $-\Delta S_{e,T}=0$ also is nearly the same, occurring at $T\sim 45$ K, see inset of Fig. 7a. Remarkably, the maxima of $-\Delta S_{e,T}$ increase almost twice along with higher temperatures in the ABA-NNL model. This is because the *L* function at $\mu =0$ captures a maximum of the reference DOS [i.e., $D(V_{g}=0)$] for the NNL-ABA model, whereas in the NL-ABA case the *L* function takes a minimum. The inversion temperature in the ABA-NNL model varies with moderate gate fields values, within the range $56\ {\text {K}} \le T \le 69$ K. As the temperature increases, $-\Delta S_{e,T}$ increase up to room temperature, cooling more for $V_g=0.4$ eV. The temperatures for $-\Delta S_{e,T}=0$ in the ABA-NNL system can also be obtained in zone II of Fig. 9a, where the reference entropy (black line) intersects with all other entropies.

**Figure 8:**
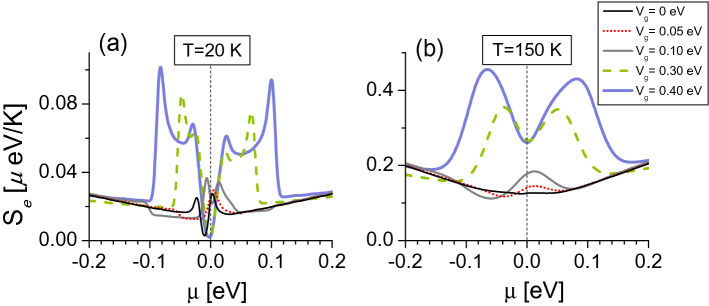
Electronic entropies as a function of $\mu$ for ABA-stacked trilayer graphene considering the NNL-TB model. (**a**) $T=20$ K, (**b**) $T=150$ K. Vertical dashed lines indicate $\mu =0$ eV, where we obtain the EC effect in Fig. 7b.

**Figure 9:**
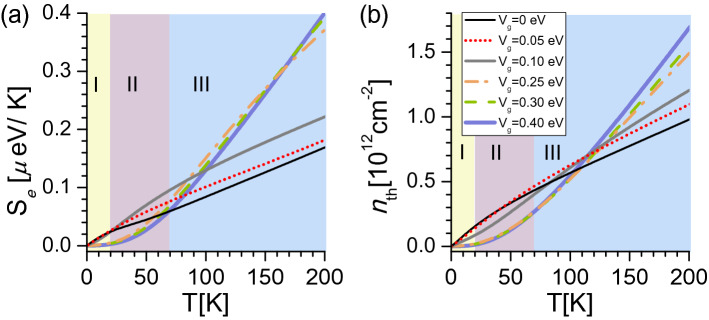
(**a**) Electronic entropy $S_e(T)$, (**b**) density of thermally excited electrons $n_{th}(T)$ versus temperature calculated at $E_\text {F}=0$ in ABA-stacked TLG within the NNL-TB model for different gate potentials $V_g$. In (**a**), I: $S_e(V_g=0) \ge S_e(V_g\ne 0)$, II: $S_e(V_g=0.05,0.1\ \text {eV})\ge S_e(V_g=0)\ge S_e(V_g=0.25,0.3,0.4\ \text {eV})$, III: $S_e(V_g=0)\le S_e(V_g\ne 0)$. Zone I, II and III in (**b**) corresponds to the same temperature range as in (**a**).

The direct-inverse response in ABA-TLG is captured by $S_{e}$ at $\mu =0$ in Fig. 8a,b. We present here only the ABA-NNL plots as both ABA system entropy results are equivalent. Figure 8a shows $S_{e}$ at $T=20$ K presents electron–hole symmetry broken, resembling the DOS shape of the ABA-NNL model in Fig. 6d. At $\mu =0$, the reference entropy $S_{e}(V_g=0)$ is lower than $S_{e}(V_g=0.05\ {\text {eV}})$, and larger than all other entropies, see Fig. 9a. Consequently, the sample cools down for $V_{g}=0.05$ eV, and heats for $V_{g}=0.1$ eV at $T=20$ K, as expected from the NNL-EC response in Fig. 7b. When the temperature increases up to $T=150$ K in Fig. 8b, $S_{e}$ is smoothed and nearly four times the entropies at $T=20$ K because of higher temperature. The reference entropy shows a flat line shape near $\mu =0$, smaller than all other entropies, indicating the system cools down for all gate fields at $T=150$ K, as also seen from the EC response.

Because of the ABA-stacked TLG is capable to heats and cools within the same sample as a direct and inverse EC response are obtained (Fig. 7), it is interesting to know how the electronic entropy $S_{e}$ is related to the charge density at the microscopic level. We calculate the number of carriers thermally excited of ABA-stacked TLG through the NNL-TB model. We determine the density of excited electrons $n_{th}$ from the valence band to the conduction band of the electronic dispersion of Fig. 6c as a function of temperature for selected values of the gate potential. In a similar way to Eq. (1), $n_{th}$ above the Fermi level at a given temperature is given by $n_{th}(V_g,T)=A^{-1} \int _{\mu }^{E_h} D(E,V_g) n_{\text {F}}(E,T,\mu ) dE$, where *A* is the unit cell area of the TLG, and we take $\mu =0$ as the Fermi energy at $T=0$ K.

The temperature dependence of $S_e(T)$ and $n_{th}(T)$ is shown in Fig. 9a,b respectively. The fraction of excited electrons follows a very similar pattern as the electronic entropy because charge carriers contribute to the entropy by filling electronic states as temperature increases. In Fig. 9b, electrons are excited at low temperature in zone I for low gate potentials $V_g=0,0.05,0.1$ eV as the electronic dispersion of the parabolic bands present negligible bandgaps at $E_{\text {F}}=0$ [see Fig. 6c,d]. As the gate potential increases, a higher temperature is needed to excite electrons from the valence band to the conduction band because of the opening of band gaps near $E_{\text {F}}=0$ in the electron dispersion. This produces a suppression (nonlinear behavior) of $n_{th}$ at low temperatures in zone I and II for moderate electric potentials $V_g=0.25,0.3,0.4$ eV. From $\sim 70$ K, $n_{th}$ linearly increases with temperature at high temperatures (zone III), which may correspond to a constant DOS as a function of energy, that is gapless parabolic electronic bands near $E_{\text {F}}$. Similar behavior for the density of excited electrons has been experimentally observed in graphene multilayers including an AB-bilayer and ABA-TLG, correlated with a temperature-dependent gap approximation, giving account of a finite-temperature electronic phase transition^50^.

Relative to our EC effect, moderate gate-field values $V_{g}=0.25,0.3,0.4$ eV induced gaps in the electronic structure of ABA-TLG, and charge carriers need a finite amount of thermal energy $k_{\text {B}}T$ to overcome those energy gaps from the valence band to the conduction band. This produces the charge suppression of $n_{th}$ at low temperatures in zone I and II of Fig. 9b. As shown in Fig. 9a in these same zones, the reference entropy is higher than the entropies for moderate gate fields, $S_e(V_g=0)>S_e(V_g=0.25,0.3,0.4)$, where occurs the direct EC effect in Fig. 7b. That is, gapped electronic states near $E_{\text {F}}$ participate in the heating mechanism of the ABA TLG system. On the contrary, the inverse EC effect is located in zone III of $S_e(T)$, where electrons are thermally excited in a linear way as a function of temperature, indicating that gapless electronic states contribute to cools down the TLG sample.

## Conclusions

Gated trilayer graphene structures present an electrocaloric response that depends on the stacking pattern and the electronic character they possess. Both the electronic structure and density of states of each TLG are very sensitive to the stacking sequence in the presence (or not) of the gate potential, especially near the Fermi level. Trilayer graphene with AAA stacking remains metallic under electrostatic gating, showing large entropy changes as the gate potential increases, linearly cooling up to room temperature. In striking contrast, ABC-stacked trilayer graphene converts into a semiconductor when the gate field is turned on, nonlinearly heating as temperature increases. The energy spectra of ABA-stacked TLG geometry present semimetallic character as well as gapped bands with moderate gate voltages, cooling and heating in the caloric response in a linear and nonlinear mixed way, giving a combination of the AAA and ABC electrocaloric effect. We verified that gate-dependent thermally excited carriers at the Fermi level for ABA-TLG can be directly linked to the electronic entropy and therefore to the EC response, where suppression of the charge carriers is related to the heating process because of entropies compensation, whereas linearly excited carriers can be associated to the cooling mechanism for moderate gate values $\ge 0.25$ eV. The advantage of heating and cooling processes under gate tunability promises interesting thermal devices within the same structure.

## Methods

### Tight-binding model

The Hamiltonians for the TLG samples ${ \widehat{{\mathscr{H}}} }^{\alpha }(\pmb {k})=\hat{H}^{\alpha }_{0}(\pmb {k})+\hat{V}$ ($\alpha ={\text {AAA,ABA,ABC}}$) include a diagonal matrix $\hat{V}$ of the gate potential proportional to $V_g$. The matrices of hopping $\hat{H}^{\alpha }_{0}(\pmb {k})$ can be obtained with the matrix elements $H_{jj'}^{\alpha }(\pmb {k})=\sum _{\pmb {R}} e^{i\pmb {k}\cdot \pmb {R}} E_{jj'}^{\alpha }(\pmb {R})$, where $E_{jj'}^{\alpha }(\pmb {R})=\left\langle \phi _{j}(\pmb {r})\right| \hat{H}_{0}^{\alpha }\left| \phi _{j'}(\pmb {r-R})\right\rangle$ is the hopping integral between the atomic orbitals $\left| \phi _{j}\right\rangle$ at $\mathbf{0}$ and $\left| \phi _{j'}\right\rangle$ at lattice vector $\pmb {R}$ with $j(j')=Ai,Bi$. $\pmb {R}_1=a_{\text {C-C}}(1,0)$, $\pmb {R}_{2(3)}=a_{\text {C-C}}\left( -1/2,+(-)\sqrt{3}/2 \right)$ are in-plane nearest-neighbor vectors with $a_{\text {C-C}}=1.42$ Å the carbon-carbon distance within a graphene layer, and $\pmb {k}=(k_x, k_y, k_z$) is the momentum. The TLG Hamiltonians are represented in the basis with components $\{\psi _{A1},\psi _{B1},\psi _{A2},\psi _{B2},\psi _{A3},\psi _{B3}\}$. Whitin this TB model, we can construct the matrix representation for each TLG structure.

The AAA-TLG Hamiltonian considering $\gamma _0$, $\gamma _1$, $\gamma _2$ and $\gamma _3$ hoppings reads

6 $$\begin{aligned} { \widehat{{\mathscr{H}}} }^{\text {AAA}}(\pmb {k})= \begin{pmatrix} -V_g &{} \gamma _0 f_{0} &{} \gamma {_1} f_{1} &{} \gamma {_3} f_{3} &{} \gamma {_2} f_{1} &{} 0 \\ \gamma _0 f_{0}^{*} &{} -V_g &{} \gamma {_3} f_{3}^{*} &{} \gamma {_1} f_{1} &{} 0 &{} \gamma {_2} f_{1} \\ \gamma {_1} f_{1}^{*} &{} \gamma {_3} f_{3} &{} 0 &{} \gamma _0 f_{0} &{} \gamma {_1} f_{1} &{} \gamma {_3} f_{3} \\ \gamma {_3} f_{3}^{*} &{} \gamma {_1} f_{1}^{*} &{} \gamma _0 f_{0}^{*} &{} 0 &{} \gamma {_3} f_{3}^{*} &{} \gamma {_1} f_{1} \\ \gamma {_2} f_{1}^{*} &{} 0 &{} \gamma {_1} f_{1}^{*} &{} \gamma {_3} f_{3} &{} V_g &{} \gamma _0 f_{0}\\ 0 &{} \gamma {_2} f_{1}^{*} &{} \gamma {_3} f_{3}^{*} &{} \gamma {_1} f_{1}^{*} &{} \gamma _0 f_{0}^{*} &{} V_g \end{pmatrix}. \end{aligned}$$

If $\gamma _{2}=\gamma _{3}=0$, and only the intralayer $\gamma _0$ and interlayer $\gamma_{1}$ hoppings are taken into account, Eq. (6) allows analytical eigenvalues because of the AAA crystal symmetry, connecting monolayer-graphene modes $\varepsilon _{1,2}$ and AA-bilayer graphenelike modes $\varepsilon _{3,4,5,6}$

7a $$\begin{aligned} \varepsilon _{1,2}&=\pm \gamma _0\left| f_0\right| , \end{aligned}$$

7b $$\begin{aligned} \varepsilon _{3,4}&=\pm \gamma _0\left| f_0\right| -\sqrt{V_g{^2}+2\gamma _{1}^{2}\left| f_1\right| ^2}, \end{aligned}$$

7c $$\begin{aligned} \varepsilon _{5,6}&=\pm \gamma _0\left| f_0\right| +\sqrt{V_g{^2}+2\gamma _{1}^{2}\left| f_1\right| ^2}. \end{aligned}$$

$f_{0}(k_x,k_y)=e^{-ik_{x}a_{\text {C-C}}}+2\cos ({\frac{\sqrt{3}}{2}k_{y}a_{\text {C-C}}})e^{ik_{x}a_{\text {C-C}}}$ and $f_{1}(k_z)=e^{i k_{z} c}$ are respectively in-plane and out-of-plane momentum-dependent functions with $c=3.3$ Å the interlayer distance^22^. The “Supplemental Material” includes density of states results for the full TB model of Eq. (6).

The TB Hamiltonian for the ABC-stacked TLG considering all the hopping interactions in Fig. 1b is given by

8 $$\begin{aligned} { \widehat{{\mathscr{H}}} }^{\text {ABC}}(\pmb {k})= \begin{pmatrix} -V_g &{} \gamma _0 f_{0} &{} \gamma _4 f_{3} &{} \gamma _3 f_{3} &{} 0 &{} \gamma _2 f_{1} \\ \gamma _0 f_{0}^{*} &{} -V_g &{} \gamma {_1} f_{1} &{} \gamma _4 f_{3} &{} 0 &{} 0 \\ \gamma _4 f_{3}^{*} &{} \gamma {_1} f_{1}^{*} &{} 0 &{} \gamma _0 f_{0} &{} \gamma _4 f_{3}^{*} &{} \gamma {_3} f_{3} \\ \gamma {_3} f_{3}^{*} &{} \gamma _4 f_{3}^{*} &{} \gamma _0 f_{0}^{*} &{} 0 &{} \gamma {_1} f_{1} &{} \gamma _4 f_{3}^{*} \\ 0 &{} 0 &{} \gamma _4 f_{3} &{} \gamma _1 f_{1}^* &{} V_g &{} \gamma _0 f_{0}\\ \gamma _2 f_{1}^{*} &{} 0 &{} \gamma {_3} f_{3}^{*} &{} \gamma _4 f_{3} &{} \gamma _0 f_{0}^{*} &{} V_g \end{pmatrix}, \end{aligned}$$

where $f_3(\pmb {k})=f_0(k_x,k_y)e^{ick_z}$. The “Supplemental Material” includes density of states and electrocaloric results for the full TB model of Eq. (8).

The TB Hamiltonian for the ABA-stacked TLG considering all hoppings in Fig. 1c

9 $$\begin{aligned} { \widehat{{\mathscr{H}}} }^{\text {ABA}}(\pmb {k})= \begin{pmatrix} -V_g &{} \gamma _0 f_{0} &{} \gamma _4 f_{3} &{} \gamma _3 f_{3} &{} \gamma _2 f_{1} &{} 0 \\ \gamma _0 f_{0}^{*} &{} -V_g &{} \gamma {_1} f_{1} &{} \gamma _4 f_{3} &{} 0 &{} \gamma _5 f_{1} \\ \gamma _4 f_{3}^{*} &{} \gamma {_1} f_{1}^{*} &{} 0 &{} \gamma _0 f_{0} &{} \gamma _4 f_{3}^{*} &{} \gamma {_1} f_{1} \\ \gamma {_3} f_{3}^{*} &{} \gamma _4 f_{3}^{*} &{} \gamma _0 f_{0}^{*} &{} 0 &{} \gamma {_3} f_{3}^{*} &{} \gamma _4 f_{3}^{*} \\ \gamma _2 f_{1}^{*} &{} 0 &{} \gamma _4 f_{3} &{} \gamma _3 f_{3} &{} V_g &{} \gamma _0 f_{0}\\ 0 &{} \gamma {_5} f_{1}^{*} &{} \gamma _1 f_{1}^{*} &{} \gamma _4 f_{3} &{} \gamma _0 f_{0}^{*} &{} V_g \end{pmatrix}. \end{aligned}$$

### Lorentzian-like function

**Figure 10:**
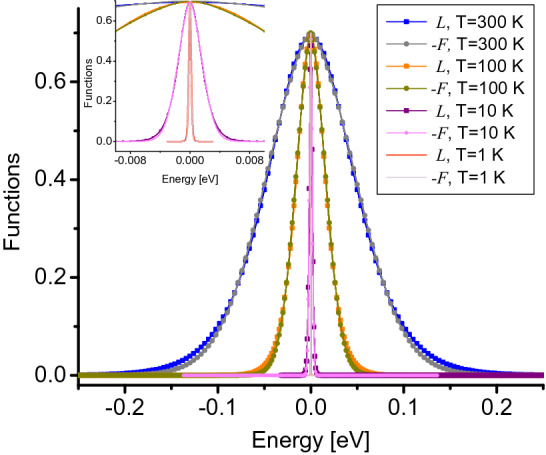
Comparison plot between the function $\mathscr {F}$ in Eq. (3) and the Lorentzian-like function *L* at $\mu =0$ eV and diverse temperatures as indicated. The inset shows a zoomed area near $E=0$ eV.

Figure 10 presents the function $\mathscr {F}$ in Eq. (3), and the Lorentzian-like function $L(E,T,\mu )=C/[e^{(|E-\mu |/2k_{\text {B}}T)^{3/2}}+1]$ we use to calculate the electronic entropy through Eq. (4) at $\mu =0$ eV in main text. Both functions $\mathscr {F}$ and *L* show excellent agreement as a function of energy at low and high temperatures.

### Tight-binding versus DFT

**Figure 11:**
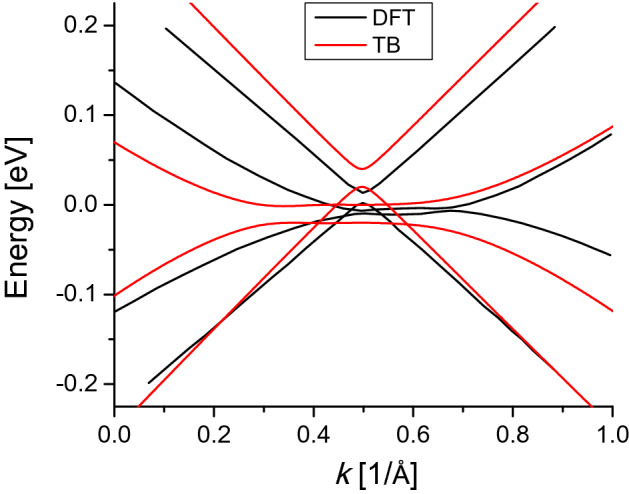
Comparison of band structure for ABA trilayer graphene. Red lines correspond to the next-nearest tight-binding model and black lines are density functional calculations^37^.

Figure 11 shows a comparison between the tight-binding model we use in our calculations and density functional band structure^37^ for ABA stacked trilayer graphene with gate potential $V_g=0$ eV. We can see a very good agreement for the whole band structure, in which both models give account of the overlap of the bands near $E_{\text {F}}=0$ eV.

## Supplementary information

Supplementary Information.
