# Supplementary material for: Gate-tunable charge carrier electrocaloric effect in trilayer graphene
Source: Sci Rep. 2021 Nov 9;11:22000. doi: 10.1038/s41598-021-01057-0 (PMC8578550; doi:10.1038/s41598-021-01057-0)
Supplement: Supplementary file 1 — Supplementary Information. [file 41598_2021_1057_MOESM1_ESM.pdf]

Supplemental Material: Gate-tunable charge carrier electrocaloric effect in trilayer graphene

Natalia Cortés[1,*], Oscar Negrete[1,2], Francisco J. Peña[1], and Patricio Vargas[1,2]

[1]Universidad Técnica Federico Santa María, Departamento de Física, Valparaíso, Casilla 110V, Chile
[2]Centro para el Desarrollo de la Nanociencia y la Nanotecnología, 8320000 Santiago, Chile
[*]natalia.cortesm@usm.cl

ABSTRACT

In this Supplemental Material, we report the density of states (DOS) for AAA and ABC stacked trilayer graphene (TLG) with next-nearest layer (NNL) hoppings, as well as electrocaloric and electronic entropy calculations for ABC TLG with NNL hoppings.

SI Next-nearest neighbors for AAA stacked trilayer graphene

Figure S1 shows the density of states for AAA stacked TLG. Panel (a) in Fig. S1 correspond to the DOS considering nearest layer (NL) γ_0 and γ_1 hoppings, as similarly seen in Fig. 2(b) of main text. Panel (b) considers additional hoppings, we have used γ_2 and γ_3 as extra interlayer hoppings[1], γ_2 connects next-nearest layer $\{Ai-Ai+2\}$ and $\{Bi-Bi+2\}$ carbon sites, and γ_3 connects nearest layer $\{Ai-Bi+1\}$ carbon sites, with i the layer index. The two main differences between both DOS in Fig. S1 is first given by the broken electron-hole symmetry about the Fermi level $E_{\mathrm{F}}=0$; second, by a small upward shift of the DOS for all gate potentials in Fig. S1(b)[1]. As the DOS for AAA TLG with NNL hoppings preserves the overall shape and metallic behavior of AAA TLG with NL hoppings about $E_{\mathrm{F}}=0$, the electrocaloric response for AAA TLG with NNL hoppings will present a very similar qualitative response as AAA TLG with NL hoppings, in which the entropy changes $-\Delta S_{e,T}$ are negative for all gate potentials we have considered, in the same way as in Fig. 3(a) of main text.

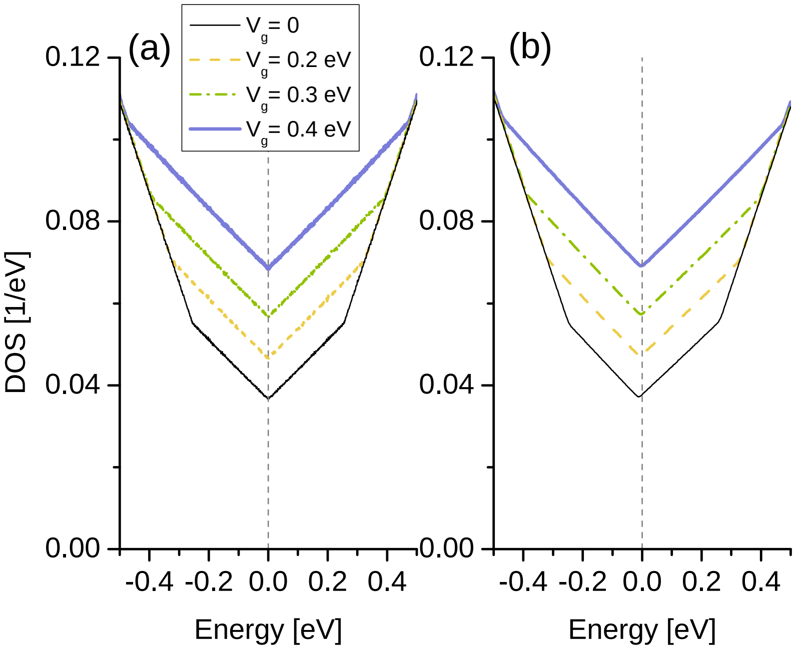

Figure S1. Density of states for AAA-stacked trilayer graphene. Panel (a) correspond to the DOS calculations with γ_0 and γ_1 hoppings as in Fig. 2(b) of main text. Panel (b) shows the DOS considering $\gamma_0=3.2$ eV, $\gamma_1=0.18$ eV[2], $\gamma_2=0.013$ eV and $\gamma_3=-0.032$ eV[1] hoppings (see Fig. 1(a) of main text for the hoppings arrange). Vertical black dashed lines indicate the Fermi level $E_{\mathrm{F}}=0$ eV.

SII Next-nearest neighbors for ABC stacked trilayer graphene

Figure S2 shows the density of states for ABC-stacked TLG considering γ_0, γ_1, γ_2, γ_3 and γ_4 couplings. The extra hopping γ_2 connects $\{Ai - Bi + 2\}$ NNL carbon sites, while γ_4 connects $\{Ai - Ai + 1\}$ and $\{Bi + Bi + 1\}$ NL carbon sites[3]. In Fig. S2, an evident difference is seen for the gate potential $V_g = 0$ by comparison to the DOS for NL hoppings of Fig. 4(b) in main text. The NNL DOS (Fig. S2) shows a minimum at the Fermi level $E_F = 0$ eV, while the NL DOS present a maximum (Fig. 4(b) of main text). Furthermore, the electron-hole symmetry is broken for the NNL DOS because of the γ_2 coupling[3]. As the DOS for $V_g = 0$ is a fundamental quantity to obtain the entropy changes (the electrocaloric effect), we have calculated $-\Delta S_{e,T}$ for the ABC-stacked TLG considering the NNL DOS of Fig. S2. Figure S3(a) shows $-\Delta S_{e,T}$ for the same V_g values as considered in Fig. 5(a) of main text. Clearly here for NNL hoppings, $-\Delta S_{e,T}$ is suppressed up to $\simeq 30$ K because of the minimum of the DOS for $V_g = 0$ at $E_F = 0$ in Fig. S2, while the opposite is seen for the NL electrocaloric effect in Fig. 5(a) of main text. Above $\simeq 30$ K, the overall behavior of $-\Delta S_{e,T}$ for all gate potentials follows the same tendency as for the NL electrocaloric effect, indicating that the extra γ_2 and γ_4 couplings have a dominant effect at low temperatures for the ABC-stacked EC effect at $E_F = 0$.

Figure S3(a) and S3(b) show the electronic entropy S_e as a function of the chemical potential μ for temperatures of $T = 30$ K and $T = 300$ K respectively. For $T = 30$ K, S_e present a very similar shape as the DOS in Fig. S2, as also seen for the NL case in Fig. 5(b) of the main text. At room temperature in Fig. S3(b), S_e is smoothed for all V_g, and for $V_g = 0.4$ eV, the electron-hole symmetry tends to be recovered.

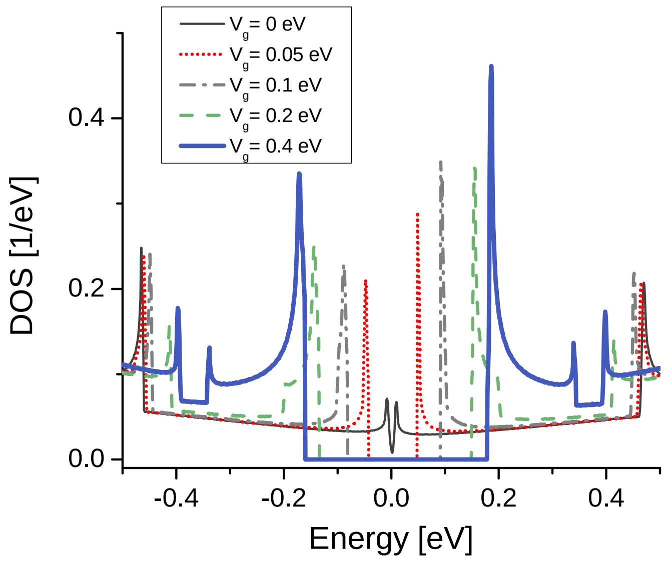

Figure S2. Density of states for ABC-stacked trilayer graphene with hoppings $\gamma_0 = 3.16$ eV, $\gamma_1 = 0.502$ eV, $\gamma_2 = -0.0171$ eV, $\gamma_3 = -0.377$ eV and $\gamma_4 = -0.099$ eV[4], see Fig. 1(b) of main text for the hoppings arrangement.

All in all, we can argue that the inclusion of additional hoppings for AAA-stacked TLG (γ_2 and γ_3) and ABC-stacked TLG (γ_2 and γ_4) does not qualitative modify the electronic entropy and electrocaloric effect results when only γ_0, γ_1 and γ_3 couplings are considered in the DOS calculations as in the main text.

References

1. L. Lu, C. *et al.* Electronic properties of AA-and ABC-stacked few-layer graphites. *J. Phys. Soc. Jpn.* **76**, 024701 (2007). DOI https://doi.org/10.1143/JPSJ.76.024701.
2. Bao, C. *et al.* Stacking-dependent electronic structure of trilayer graphene resolved by nanospot angle-resolved photoemission spectroscopy. *Nano Lett.* **17**, 1564–1568 (2017). DOI https://doi.org/10.1021/acs.nanolett.6b04698.
3. Koshino, M. & McCann, E. Trigonal warping and berry's phase N π in ABC-stacked multilayer graphene. *Phys. Rev. B* **80**, 165409 (2009). DOI https://doi.org/10.1103/PhysRevB.80.165409.
4. Zhang, F., Sahu, B., Min, H. & MacDonald, A. H. Band structure of ABC-stacked graphene trilayers. *Phys. Rev. B* **82**, 035409 (2010). DOI https://doi.org/10.1103/PhysRevB.82.035409.

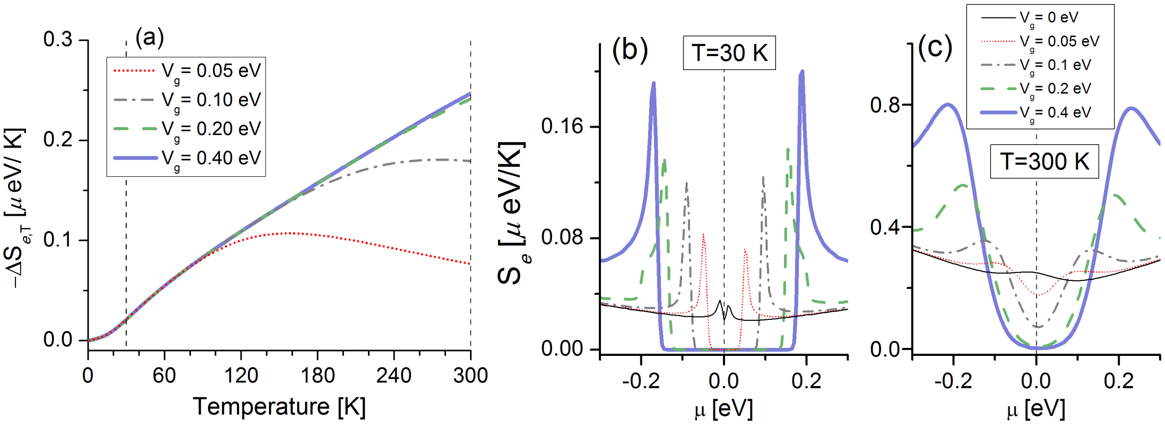

Figure S3. (a) Electrocaloric effect for ABC-stacked trilayer graphene including γ_2 and γ_4 hoppings at $E_F = 0$. The electronic entropy as a function of μ for (b) $T = 30$ K and (c) $T = 30$ K. Dashed vertical lines in (a) indicate the temperatures where S_e is calculated in panels (b) and (c). In (b) and (c) $E_F = 0$ is indicted with a dashed vertical line.
